# Comparative Analysis Reveals Distinct and Overlapping Functions of Mef2c and Mef2d during Cardiogenesis in *Xenopus laevis*

**DOI:** 10.1371/journal.pone.0087294

**Published:** 2014-01-28

**Authors:** Yanchun Guo, Susanne J. Kühl, Astrid S. Pfister, Wiebke Cizelsky, Stephanie Denk, Laura Beer-Molz, Michael Kühl

**Affiliations:** 1 Institute of Biochemistry and Molecular Biology, Ulm University, Ulm, Germany; 2 International Graduate School of Molecular Medicine Ulm, Ulm University, Ulm, Germany; University of Miami School of Medicine, United States of America

## Abstract

The family of vertebrate Mef2 transcription factors is comprised of four members named Mef2a, Mef2b, Mef2c, and Mef2d. These transcription factors are regulators of the myogenic programs with crucial roles in development of skeletal, cardiac and smooth muscle cells. Mef2a and Mef2c are essential for cardiac development in mice. In *Xenopus*, *mef2c* and *mef2d* but not *mef2a* were recently shown to be expressed during cardiogenesis. We here investigated the function of Mef2c and Mef2d during *Xenopus laevis* cardiogenesis. Knocking down either gene by corresponding antisense morpholino oligonucleotides led to profound heart defects including morphological abnormalities, pericardial edema, and brachycardia. Marker gene expression analyses and rescue experiments revealed that (i) both genes are required for proper cardiac gene expression, (ii) Mef2d can compensate for the loss of Mef2c but not *vice versa*, and (iii) the γ domain of Mef2c is required for early cardiac development. Taken together, our data provide novel insights into the function of Mef2 during cardiogenesis, highlight evolutionary differences between species and might have an impact on attempts of direct reprogramming.

## Introduction

In the mouse, the heart arises from two populations of cells referred to as the first and second heart field, respectively [1]–[3]. Cells of the first heart field (FHF) localize in the cardiac crescent and later form the linear heart tube that grows by continuous addition of cells from the venous as well as the arterial pole. These newly added cardiac cells originate from the so-called second heart field (SHF) that is characterized by the expression of transcription factors Islet-1 (*Isl1*) and T-box factor 1 (*Tbx1*) [4]. From lineage labeling studies, it is known that cells of the SHF contribute to most of the right ventricle and the outflow tract and contribute to the aortic sac, the endocardium, the ventricular and atrial septum as well as to the atrial myocardium whereas the other parts of the heart are mainly derived from cells of the FHF [5]. Data gained from work in mouse ES cells suggest that the FHF and the SHF are established from a common cardiac progenitor cell (CPC) population.

Cardiac development in *Xenopus* starts at the onset of gastrulation with the induction of two bilaterally located cardiac progenitor cell populations on either side of the Spemann Organizer [6], [7]. During gastrulation, these cells migrate towards the anterior-ventral side of the embryo and fuse to a single common CPC population located adjacent and posterior to the cement gland anlage [8]. During further development, this CPC population becomes more and more heterogenic, so that at stage 24, two populations of cells can be distinguished by differential gene expression. Lineage labeling experiments have revealed that these two separate cell populations correlate to the first and the second heart field in mice, respectively. At this stage, cells of the *Xenopus* FHF already express genes indicating cardiac differentiation such as cardiac troponin (*tnni3*) and myosin heavy chain (*myh6*). Upon further development, these cells will later contribute to forming the single ventricle, the two atria and the inflow tract [8]. Cells of the SHF are localized anterior to the first heart field adjacent to the cement gland and express *isl1* and *tbx1* as well as bone morphogenetic protein 4 (*bmp4*). These cells have been shown to develop into the outflow tract [8].

Mef2 (myocyte enhancer factor 2) proteins belong to the MADS (MCM1, agamous, deficient, serum response factor) family of transcription factors. Members of the Mef2 family contain a common MADS-box and a Mef2-type domain at their N-terminus. The MADS-box serves as a minimal DNA-binding domain that requires the adjacent Mef2-type domain for dimerization and high-affinity DNA binding [9]–[11]. These proteins also contain a transactivation domain at the C-terminus which is involved in the regulation of transcriptional activity. The four vertebrate Mef2 genes - referred to as *Mef2a*, *b*, *c* and *d-* are expressed in precursors of the three muscle lineages as well as in neurons [10]. The vertebrate Mef2 gene products share about 50% overall amino acid identity and are about 95% similar throughout the highly conserved MADS-box and Mef2-type domain. In contrast, they are more divergent in their C-terminal regions [10], [11]. Additionally, each *Mef2* gene gives rise to multiple isoforms through alternative splicing that are expressed in different embryonic and adult tissues [12]. In mammals, three major exons are alternatively spliced in *Mef2* genes: the two mutually exclusive exons a1 and a2, and the short exons β and γ. The function of these isoforms depends on the exon composition after splicing. In particular the amino acids coded by the γ exon have been shown to function in gene repression [12]. The predominantly expressed Mef2c isoform in the adult murine heart as well as during differentiation of mouse C2C12 into myocytes is lacking this γ domain [12]. For *Xenopus mef2c*, three exons are alternatively spliced: two exons corresponding to the mammalian β and γ exons, as well as the so-called δ exon. No *mef2d* splice variants were detected in *Xenopus* [13].

Mef2 transcription factors regulate a wide array of genes including numerous muscle-specific enzymes, structural proteins and other transcription factors, e.g. MyoD or MHCα. Loss of function mutations in the single *mef2* gene in the *Drosophila* embryo result in an abnormal development of all muscle cell types including cardiomyocytes [14]. In mouse, *Mef2a*, *c* and *d* are detected in the cardiac mesoderm [15]. Mef2d knock out mice do not exhibit any obvious embryonic phenotypes and a Mef2b knock out mouse has not yet been generated. In contrast, Mef2a or Mef2c null mice show defects in cardiac development [16], [17]. In particular, Mef2a knock out mice die perinatally from an array of heart defects including severe cardiac cytoarchitectural defects and right ventricular chamber dilation [18]. Mef2c null mice die at embryonic day 10.5 due to failure in cardiac development, e.g. cardiac edema, defects in cardiac looping, brachycardia and deficits in the development of the right ventricle. The animals also exhibit vascular defects [17], [19]–[21]. Meanwhile, *Mef2c* has been integrated into a complex gene regulatory network acting through early cardiogenesis [22]. The important role of Mef2c during cardiac development was also recently demonstrated by showing that Mef2c together with other transcription factors can cooperatively reprogram adult mouse tail-tip and cardiac fibroblasts into beating cardiomyocyte-like cells *in vitro* and *in vivo* [23]–[25]. In adult mice, Mef2a and Mef2d represent the major isoforms expressed [26]. Gain and loss of function studies in genetically modified mice indicated an important function of Mef2d for stress-dependent cardiac growth. Overexpression of Mef2d also resulted in reactivation of fetal cardiac gene expression in the adult heart [26], whereas loss of Mef2a in contrast is lethal [18]. Understanding the molecular function of these transcription factors in more detail is thus crucial for gaining deeper insights into cardiac development as well as cell reprogramming.

*mef2c* and *mef2d* expression was recently shown in cardiac tissue of *Xenopus laevis* embryos whereas *mef2a* is not expressed in the heart [8], [13], suggesting a role of Mef2c and Mef2d during *Xenopus* heart development. This is in contrast to the situation in mouse where, as discussed above, Mef2a and Mef2c but not Mef2d knock out mice display an embryonic cardiac phenotype. By loss of function studies, we show here for the first time that both Mef2c and Mef2d are required for cardiogenesis in *Xenopus*. In addition, knocking down Mef2c or Mef2d leads to a down-regulation of characteristic genes in both, the first and second heart field. Furthermore, we observed that depletion of Mef2c can be rescued by Mef2c and MEF2D, while Mef2c cannot compensate for the loss of Mef2d in *Xenopus* embryos. Equally important, we also show for the first time that the γ domain of Mef2c is essential for correct cardiac development.

## Results

### *mef2c* and *mef2d* are expressed in the developing heart in *Xenopus laevis*

**Figure 1:**
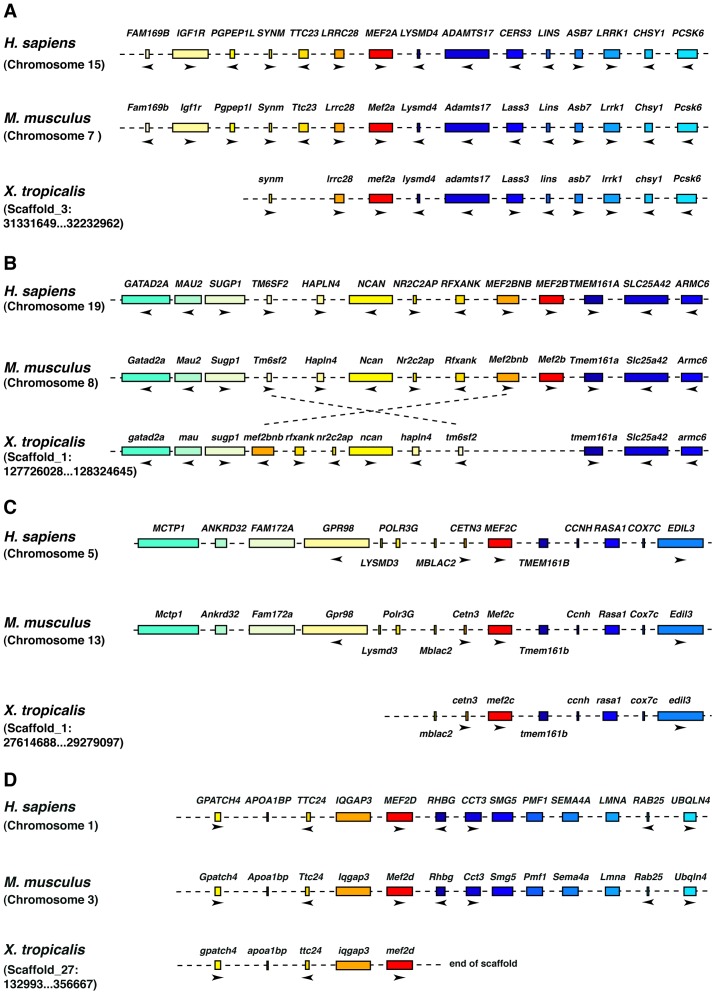
Synteny analyses of Mef2a, b, c, and d. **A.** Synteny analysis of *mef2a.* Schematic overview comparing the *mef2a* gene *in Homo sapiens* (chromosome 15), *Mus musculus* (chromosome 7) and *Xenopus tropicalis* (scaffold_3, Xenbase G-Browse). **B.** Synteny analysis of *mef2b*. Schematic overview comparing the mef2b gene *in Homo sapiens* (chromosome 19), *Mus musculus* (chromosome 8) and the *mef2b* neighboring genes in *Xenopus tropicalis* (scaffold_1, Xenbase G-Browse). **C.** Synteny analysis of *mef2c*. Schematic overview comparing the *mef2c* gene *in Homo sapiens* (chromosome 5), *Mus musculus* (chromosome 13) and *Xenopus tropicalis* (scaffold_1, Xenbase G-Browse). **D.** Synteny analysis of *mef2d*. Schematic overview comparing the mef2d gene *in Homo sapiens* (chromosome 1), *Mus musculus* (chromosome 3) and *Xenopus tropicalis* (scaffold_27, Xenbase G-Browse). In all panels conserved genes are indicated by a color code. The orientation of the open reading frames of some genes is depicted by arrowheads. Gene length or distances between genes are not drawn to scale. A list of gene abbreviations used here is given in Table S1.

To initiate this study we first analyzed available sequence information on *mef2* genes in *Xenopus laevis* and *Xenopus tropicalis*. Comparing the synteny of the *Mef2a*, *Mef2c*, and *Mef2d* genes in the human and the mouse genomes with the corresponding homologs in *Xenopus* confirmed that the available genomic sequences and EST clones code for the corresponding homologs in *Xenopus* (Fig. 1 A, C and D, Table S1) and that the current annotations of these *mef2* family members in *Xenopus* are correct. For *mef2b*, no EST sequences were reported so far and no homolog has been identified in the *Xenopus tropicalis* genome. Therefore, we analyzed the genomic region of the *Xenopus tropicalis* genome where *mef2b* should be localized in more detail. In humans, *Mef2b* is localized on chromosome 19 and the neighboring genes are well known. This synteny is well conserved between the human and the mouse genomes (Fig. 1 B, Table S1). Using this information, we were able to identify one scaffold that should contain the genomic information for *Xenopus mef2b*. Whereas the orthologs of the *mef2b* flanking genes could be identified on this scaffold, no sequences coding for *mef2b* were found. Interestingly, a region spanning about 277 kb was inverted in comparison to the human and mouse genomes. One DNA break point required for this inversion covers the region where *mef2b* should be located. This observation and the lack of any *mef2b* EST suggest that the gene coding for *mef2b* was lost in *Xenopus* during evolution.

RT-PCR experiments revealed that *Xenopus mef2c* and *mef2d* transcripts are maternally supplied and expressed during the entire embryogenesis (Figure S1 A, B). We next confirmed the spatio-temporal expression pattern of *mef2c* and *mef2d* during *Xenopus laevis* cardiac development as previously published [8], [13]. *Xenopus mef2c* transcripts were faintly detected in the common cardiac progenitor cell (CPC) population at stage 20 whereas *mef2d* transcripts were strongly detected in CPCs (Figure S1 C, L). During further development, *mef2c* and *mef2d* are continuously expressed in cardiac tissue (Figure S1 D–M, K–T). At stages 28 and later, both genes are expressed in the first heart field (FHF) region (Figure S1 F–K, O–T), whereas only *mef2c* is expressed in the more lateral region of the second heart field (SHF) (Figure S1 G, red arrowhead). Transverse sections revealed that *mef2c* is expressed in the myocardium (arrowhead) as well as in the endocardium (arrow) during tailbud stages (Figure S1 h), while stronger *mef2d* expression is restricted to the myocardium at this time point (Figure S1 q, arrow). At stage 36, expression of both, *mef2c* and *mef2d*, was observed in the myocardium (Figure S1 j, s, arrows).

### Loss of either Mef2c or Mef2d results in cardiac malformations

For loss of function studies we relied on morpholino oligonucleotide (MO) based antisense strategies. To test the binding efficiency and functionality of the MOs used, the MO binding sites of *Xenopus mef2c* (here denoted as *xmef2c*), and *Xenopus mef2d* (*xmef2d*) were cloned into the pCS2+ expression vector in front of and in frame with the *GFP* gene. The RNAs of these constructs were bilaterally co-injected with either Control MO or Mef2c MO or Mef2d MO into two-cell stage embryos. GFP expression was monitored at stage 20 (Figure S2). Co-injection of the *xmef2c-GFP* fusion construct together with Mef2c MO blocked the translation of GFP, whereas co-injection of a Control MO had no effect on the expression. Similar, co-injection of the *xmef2d-GFP* fusion construct together with Mef2d MO also blocked the translation of GFP. In order to rescue the observed phenotypes seen after knocking down either Mef2c or Mef2d, we relied on RNA coding for murine *Mef2c* or human *MEF2D* that were not targeted by either MO due to sequence differences in the 5′UTR (*mMef2c*) or due to introduced silent point mutations in the coding sequence (*hMEF2D*). Again, the efficiency was tested as described above. The expression of *mMef2c-GFP* or *hMEF2D-GFP* was not blocked by Mef2c MO. The Mef2d MO did not have an inhibitory effect on *hMEF2D-GFP* or *mMef2c-GFP* (Figure S2). Taken together, these results support the idea that (I) both MOs specifically block the translation of the corresponding *Xenopus mef2* gene, (II) Mef2c MO neither inhibits mMef2c nor hMEF2D translation and (III) the Mef2d MO does not interfere with mMef2c or hMEF2D expression. In conclusion, RNA coding for murine *Mef2c* and human *MEF2D* can be used as gene specific rescue constructs.

**Figure 2:**
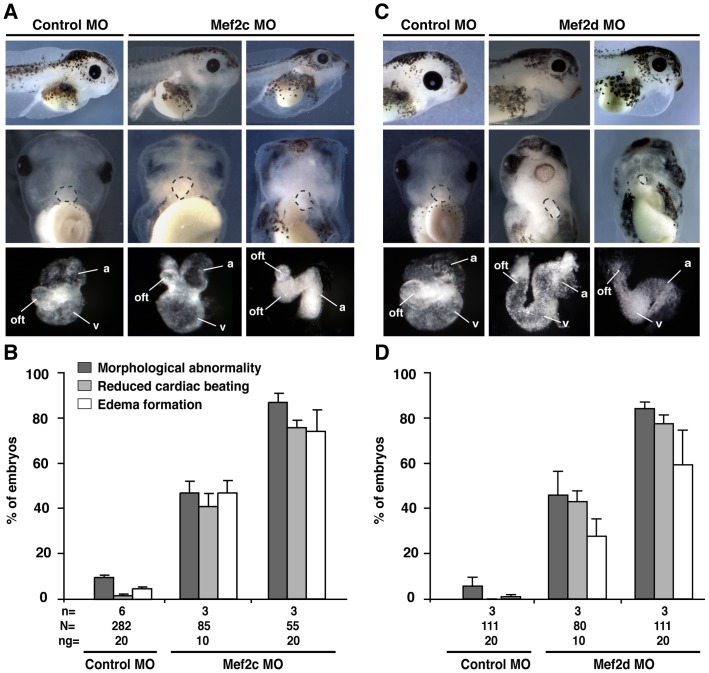
Depletion of either *mef2c* or *mef2d* leads to severe cardiac defects. **A, C.** Mef2c- or Mef2d MO-injected embryos developed cardiac edema and morphological heart defects at stage 42, as compared with Control MO-injected sibling embryos. The amount of MO injected in total (two blastomeres) is given in ng. In addition, cardiac beating was reduced upon Mef2c or Mef2d MO depletion. The dotted dark gray lines indicate the heart. a: atria; v: ventricle; oft: outflow tract. **B, D.** Quantitative presentations are shown. N: number of examined embryos; n: number of independent experiments.

To interfere with Mef2c or Mef2d function during heart formation, either Mef2c or Mef2d MOs were injected bilaterally into both dorso-vegetal blastomeres of eight-cell stage *Xenopus* embryos to target cardiac tissue [27]. In all experiments, 0.5 ng *GFP* RNA was co-injected as a lineage tracer and to identify correctly injected embryos. Mef2c and Mef2d morphant embryos revealed abnormalities during cardiogenesis whereas Control MO-injected siblings showed a normal cardiac development (Fig. 2 A–D). Embryos depleted of Mef2c or Mef2d developed pericardial edema and malformed hearts and additionally showed a reduced heart rate at stage 42 (Fig. 2A, B). To take a closer look at morphant hearts, we isolated the hearts of fixed *Xenopus* embryos. We observed that the heart tube was formed and the looping process was initiated in both Mef2c and Mef2d morphant embryos. However, during later development, defects during the looping process as well as smaller cardiac chambers were observed in Mef2c and Mef2d MO injected embryos (Figure 2 A–D). These results indicate that both members of the Mef2 family are required for cardiogenesis in *Xenopus*, and are particularly important for cardiac morphogenesis and heart looping.

### Mef2c or Mef2d are required for cardiac gene expression in the common cardiac progenitor cell population

**Figure 3:**
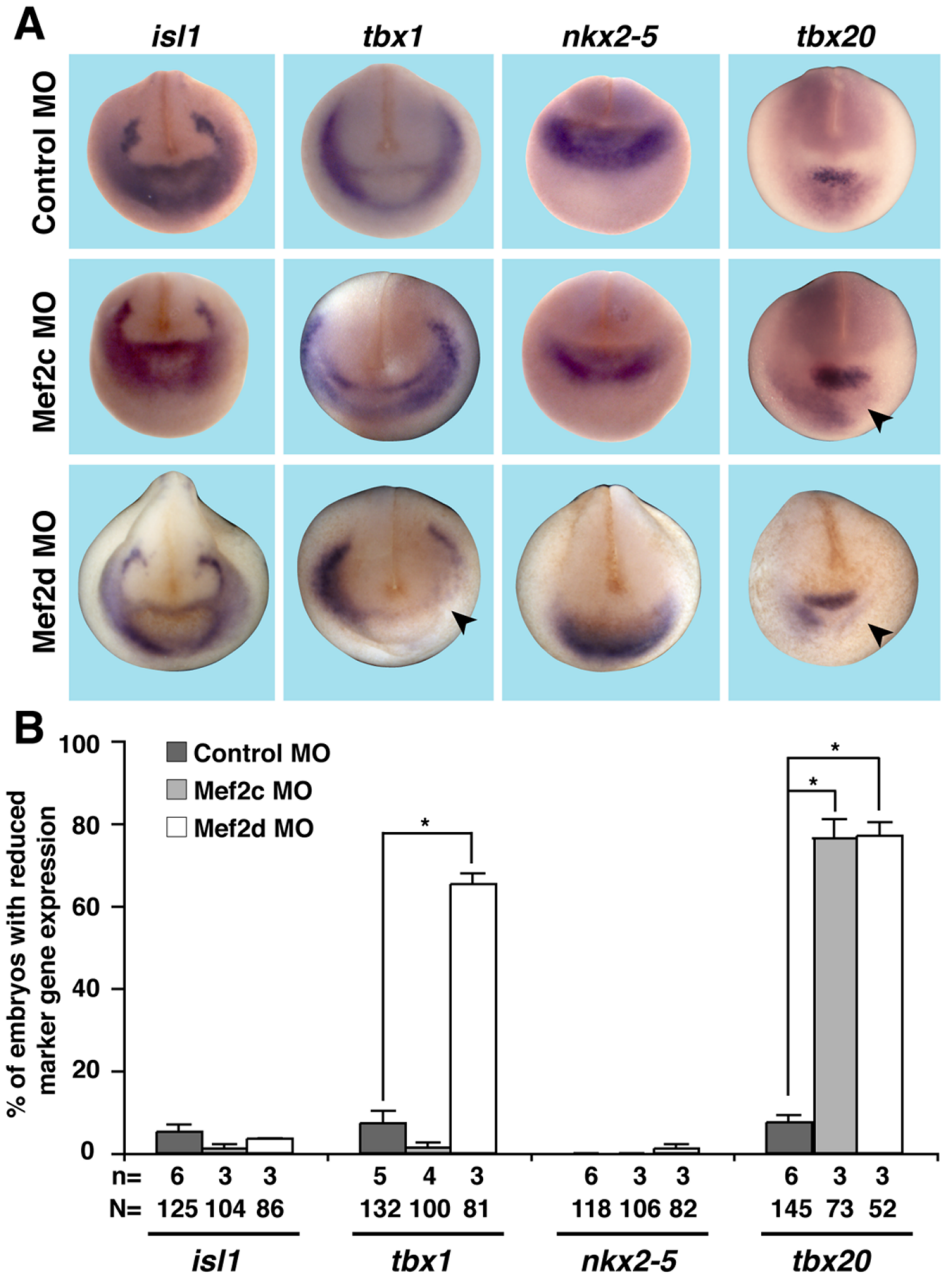
Loss of Mef2c or Mef2d affects the cardiac progenitor cell population. Mef2c or Mef2d MO (10 ng) was unilaterally injected and expression of cardiac marker genes was monitored at stage 20. **A.** *tbx20* expression was down-regulated in Mef2c MO- or Mef2d MO-injected embryos (arrowheads). In addition, Mef2d- but not Mef2c-depleted embryos showed reduced *tbx1* expression (arrowhead). Expression of *isl1* as well as *bmp4* remained unchanged upon loss of Mef2c or Mef2d. Anterior views of embryos are shown. **B.** Quantitative presentation of observed phenotypes in A is given. N: number of examined embryos; n: number of independent experiments; *, *p*≤0.05.

Mef2 transcription factors have been shown to be important regulators of cardiac differentiation [14], [16], [17]. To pinpoint the time point of when a loss of either Mef2 factor affects cardiogenesis, we analyzed the expression of different cardiac marker genes at different time points of cardiogenesis. For this purpose, we injected Mef2c or Mef2d MO unilaterally into one dorso-vegetal blastomere of eight-cell stage *Xenopus* embryos and analyzed marker gene expression at different stages by whole mount *in situ* hybridization. In this experimental setting, the uninjected side served as an internal control. The injected side was identified by co-injecting *GFP* RNA as a lineage tracer. We started our analysis at stage 20 when the CPC population is located on the ventral side of the embryo [8]. At this stage, the expression of *tbx20* was found to be down-regulated on the injected side of Mef2c or Mef2d-depleted embryos as compared to the uninjected side and Control MO-injected siblings (Fig. 3). Loss of Mef2d but not Mef2c reduced *tbx1* expression at this stage. The expression of *isl1* and *nkx2-5* however was not affected in either Mef2c or Mef2d deficient embryos (Fig. 3). This observation indicates that both Mef2c and Mef2d are required for proper expression of cardiac genes in the *Xenopus* CPC population.

**Figure 4:**
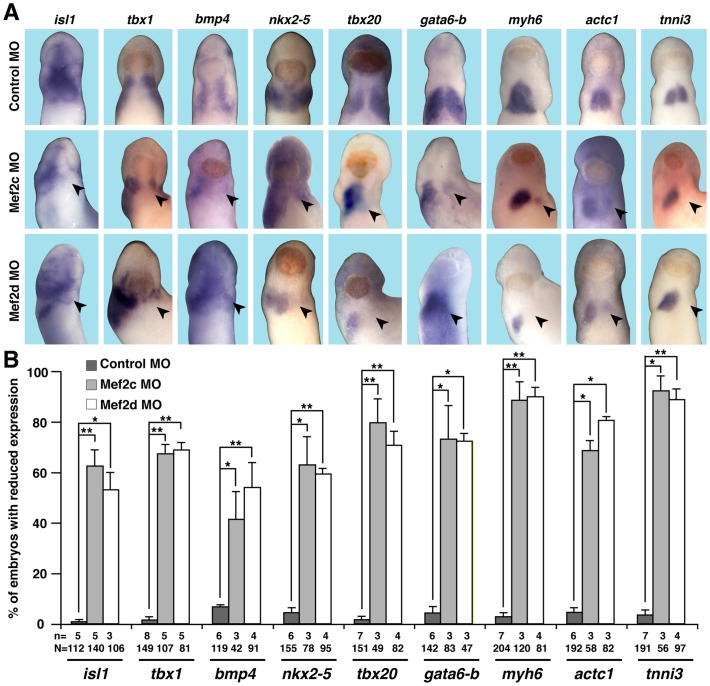
Cardiac differentiation is affected upon loss of Mef2c or Mef2d. **A.** At stage 28, depletion of Mef2c or Mef2d led to a reduced expression of cardiac markers including *isl1*, *bmp4*, *nkx2.5*, *tbx1*, *tbx20*, *gata6b*, *myh6*, *actc1*, and *tnni3* (arrowheads). Ventral views of embryos are shown. **B.** Quantitative presentation of the observed phenotype in A is given. N = number of examined embryos; n = number of independent experiments; *, *p*≤0.05; **, *p*≤0.01.

At stage 28, when the CPC population has split into the FHF and SHF [8], both Mef2c MO and Mef2d MO-injected embryos revealed reduced expression of FHF marker genes such as *tbx20* and *gata6b* as well as SHF marker genes such as *tbx1* and *isl1*. Marker genes indicating terminal differentiation of cardiomyocytes, namely *actc1*, *myh6* and *tnni3*, were also down-regulated upon loss of either gene (Fig. 4).

### The γ domain of Mef2c is required for embryonic cardiogenesis

**Figure 5:**
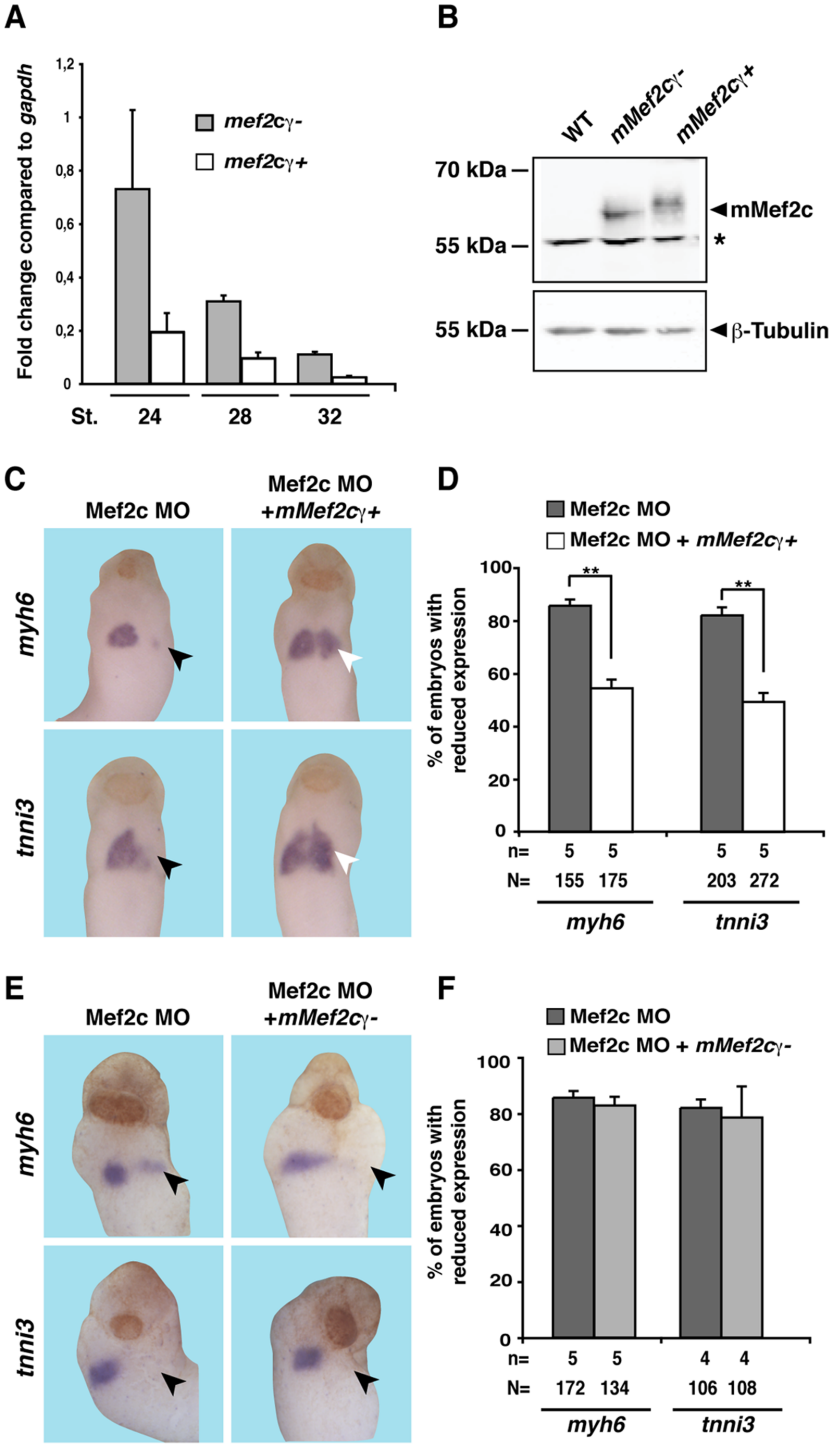
Specificity of phenotype observed upon knock down of Mef2c. **A.** *mef2c*γ- and *mef2c*γ+ isoforms are expressed on RNA level in heart tissue enriched explants at stages 24, 28, and 32 as revealed by qPCR. Expression is shown relative to *gapdh*. **B.** mMef2cγ- and mMef2cγ+ are expressed on protein level upon RNA injection into *Xenopus* embryo at comparable levels. β-Tubulin served as loading control. Note that the Mef2c antibody used does not recognize endogenous *Xenopus* Mef2c protein. The asterisk indicates unspecific background. **C–F.** Mef2c MO was unilaterally injected together with *GFP*, *mMef2c*γ+ *or mMef2*γ- RNA as indicated. ***C, E*** Expression of the cardiac marker genes *myh6* and *tnni3* was monitored at stage 20 or stage 28. Black arrowheads indicate reduced marker gene expression, white arrowheads highlight the rescued situation. Ventral views of embryos are shown. **D, F.** Quantitative presentations are shown. N: number of examined embryos; n: number of independent experiments; st: stage; **, *p*≤0.01.

**Figure 6:**
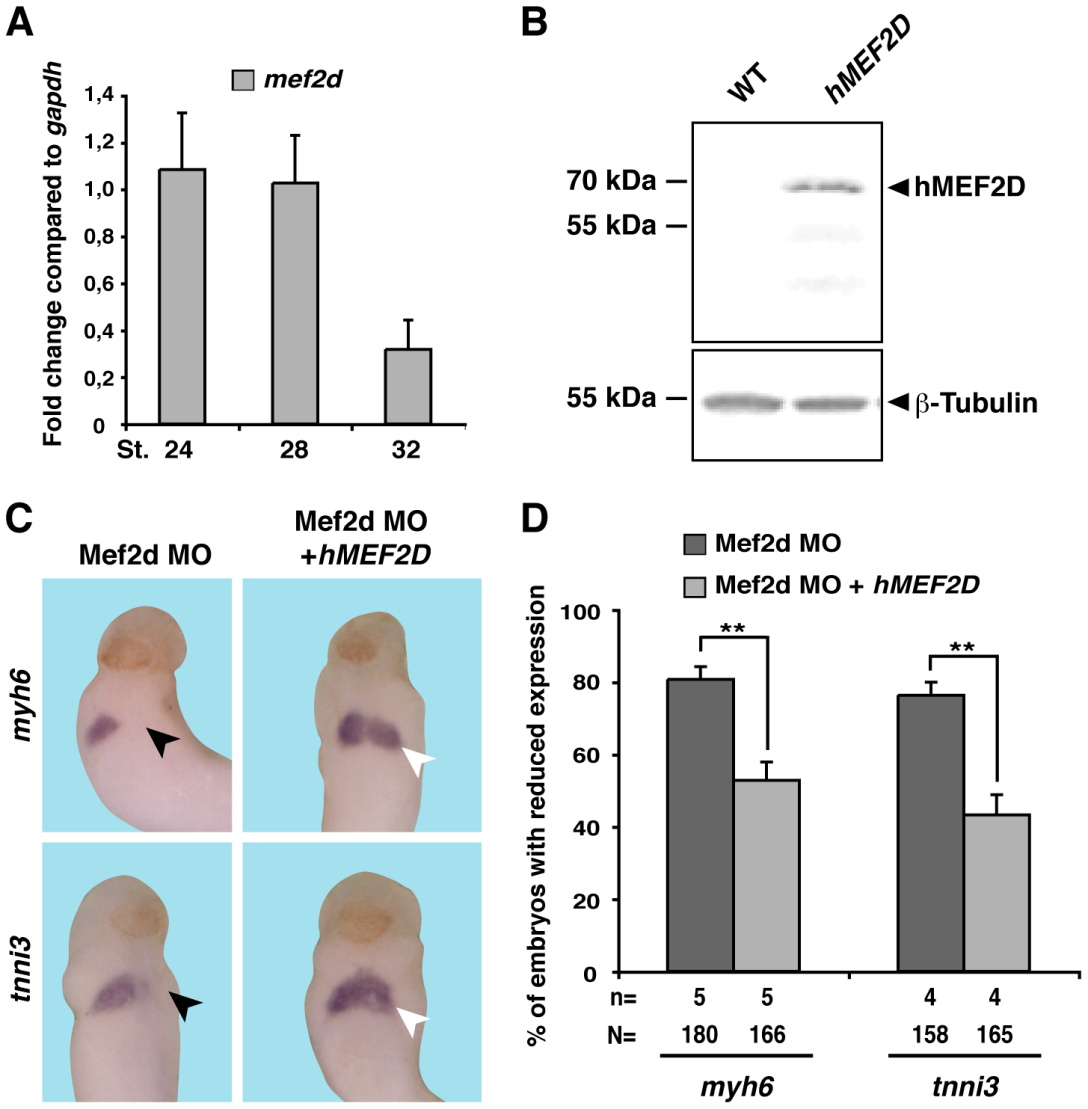
Specificity of phenotype observed upon knock down of Mef2d. **A.** *mef2d* isexpressed on RNA level in heart tissue enriched explants at stages 24, 28, and 32 as revealed by qPCR. Expression is shown relative to *gapdh*. **B.** hMEF2D is expressed on protein level upon RNA injection into *Xenopus* embryo. β-tubulin served as loading control. Note the Mef2d antibody used does not recognize endogenous *Xenopus* Mef2d protein. **C.** Mef2d MO was unilaterally injected together with *GFP* or *hMEF2D as indicated*. Expression of cardiac marker genes *myh6* and *tnni3* was monitored at 28. Black arrowheads indicate reduced marker gene expression, white arrowheads highlight the rescued situation. **D.** A quantitative presentation of results is given. N: number of examined embryos; n: number of independent experiments; **, *p*≤0.01.

While cloning the *Xenopus laevis mef2c* gene from heart enriched explants, we isolated two different splice versions of this gene that differ in either the presence or absence of the γ domain, *mef2c*γ- and *mef2c*γ+, respectively. qPCR analyses with primer pairs specifically recognizing either splice variant revealed that both isoforms of Mef2c are expressed in heart tissue enriched explants cut at stages 24, 28, and 32 (Fig. 5 A). Please note that cardiac explants cannot be cut at stage 20 of development as the cardiac tissue at this stage is a thin layer of cells underlying the ectoderm in the anterior region of the embryo in close proximity to other sites of Mef2 expression. Interestingly, *mef2c*γ- was expressed at higher levels on RNA level than the *mef2c*γ+ variant. This is in agreement with previous data showing that *mef2c*γ- is the predominantly expressed variant during murine embryogenesis and the only isoform that was found to be expressed in the murine adult heart [12]. Moreover, also *mef2d* could be detected by qPCR experiments. (Fig. 6A).

We next aimed to show the specificity of the observed phenotypes. To generate a *mMef2c*γ- construct, we deleted this domain in the m*Mef2c*γ+ construct. Western Blot experiments using a Mef2c antibody showed that both variants of mMef2c are expressed at similar levels upon RNA injection (Fig. 5 B). The same holds true for the injected hMEF2D RNA which was detected by a specific MEF2D antibody (Fig. 6B). For rescue experiments, we co-injected in a first set of experiments Mef2c MO together with RNA coding for murine *Mef2c*γ+ and examined expression of *myh6* and *tnni3* at stage 28. This co-injection led to a significant rescue of marker gene expression (Fig. 5 C, D). Intriguingly, the *mMef2c*γ- version failed to rescue the observed changes in gene expression upon Mef2c knock down (Fig. 5 E, F). These data indicate for the first time that the γ domain of Mef2c has an important role during early cardiogenesis in *Xenopus*. The restored expression of *myh6* and *tnni3* was also observed in embryos co-injected with the Mef2d MO together with *hMEF2D* mRNA (Fig. 6 C, D).

### Mef2d can compensate for the loss of *mef2c* but not vice versa

**Figure 7:**
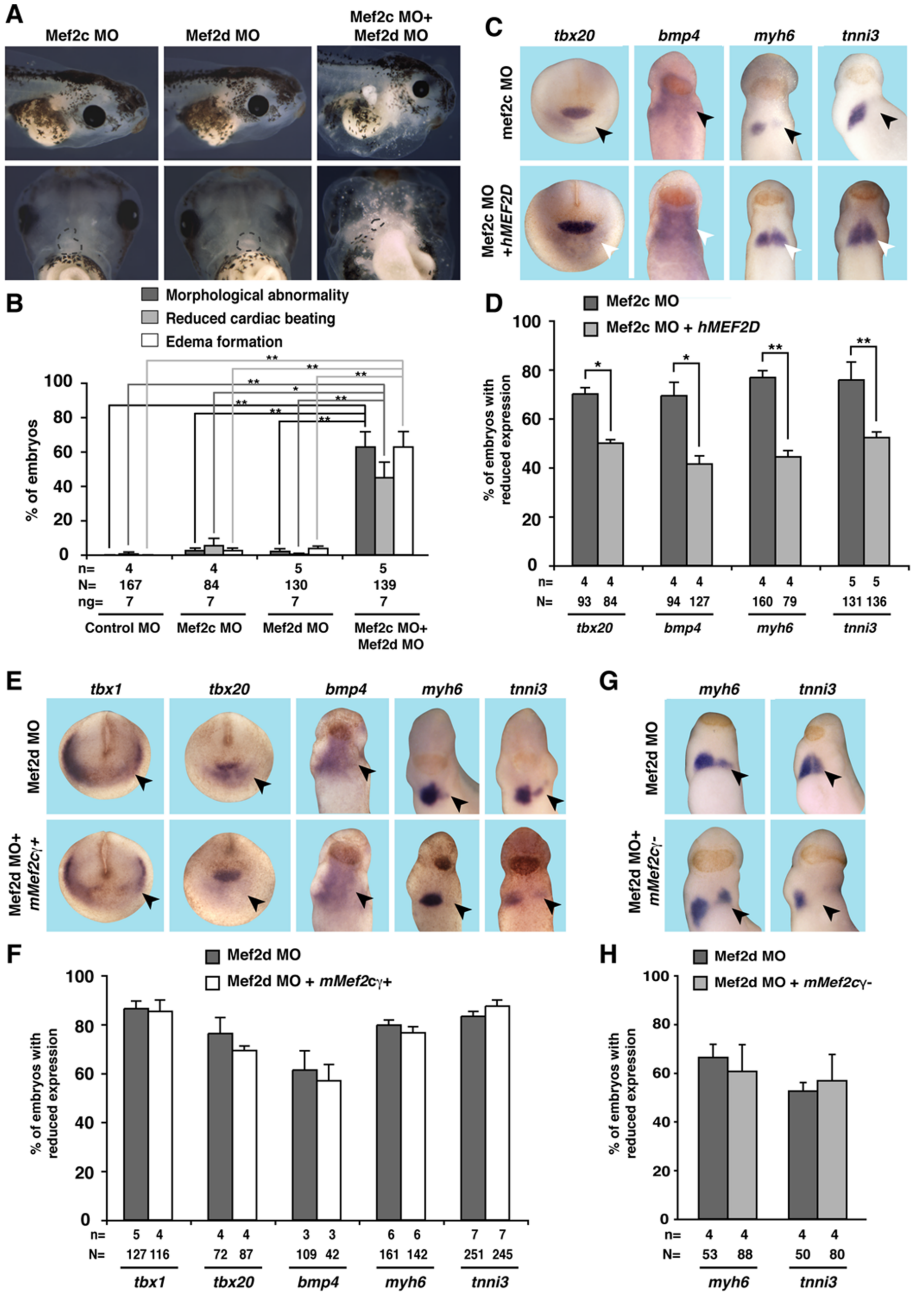
Cooperation of Mef2c and Mef2d in *Xenopus*. **A, B.** The injection of 7(in both cases 3.5 ng per blastomere) did not result in cardiac defects. The co-injection of 7 ng Mef2c together with 7 ng Mef2d MO led to a significant increase of the phenotype. The dotted black lines indicate the heart. **C, D.** Mef2c MO was unilaterally injected along with RNA coding for hMEF2D. Expression of cardiac marker genes was monitored at stages 20 (*tbx20*, *bmp4*; anterior views) or stage 28 (*myh6*, *tnni3*; ventral views), respectively. **E, F.** Mef2d MO was unilaterally injected along with *mMef2c*γ+ RNA. Expression of cardiac marker genes was monitored at stages 20 (*tbx1*, *tbx20*; anterior views) or stage 28 (*bmp4*, *myh6*, *tnni3*; ventral views), respectively. **G, H.** Mef2d MO was unilaterally injected along with *mMef2c*γ ˜ RNA. Expression of cardiac marker genes was monitored at stage 28 (ventral view). In all cases, black arrowheads indicate reduced marker gene expression, white arrowheads highlight the rescued expression. **B, D, F, H.** Quantitative presentations of the experiments shown in **C, E, G** are shown. N: number of examined embryos; n: number of independent experiments; *, *p*≤0.05; **, *p*≤0.01.

Given the similarities of the phenotypes observed upon depletion of either Mef2c or Mef2d but also taking into account the differences in the effects of either MO on gene expression in the CPC population, we aimed to further characterize the interplay of both factors. First, we injected low doses of Mef2c and Mef2d MO either alone or in combination into both dorso-vegetal blastomeres of eight-cell stage embryos (Fig. 7 A, B). Interestingly, whereas neither a low dose of Mef2c MO nor Mef2d MO resulted in a phenotype, the co-injection of both MOs at this low dose together resulted in a robust phenotype in a more than additive manner indicating a synergistic activity of both transcription factors. We furthermore performed cross-rescue experiments by co-injecting Mef2c MO together with *hMEF2D* or Mef2d MO with *mMef2c*γ or *mMef2c*γ ˜ RNA and analyzed marker gene expression at stages 20 and 28. By co-injecting the Mef2c MO together with *hMEF2D*, the expression of *tbx20* at stage 20 and *bmp4*, *myh6* and *tnni3* at stage 28 was significantly restored (Fig. 7 C, D). However, co-injection of Mef2d MO with *mMef2c* failed to rescue the observed phenotypes independent of the *mMef2c* splice variant used (Fig. 7 E–H). These data clearly indicate that Mef2d can compensate for a loss of Mef2c but not *vice versa*.

### Overexpression of *mMef2*γ+ or *hMEF2D* but not *mMef2c*γ- accelerates the onset of cardiac differentiation

**Figure 8:**
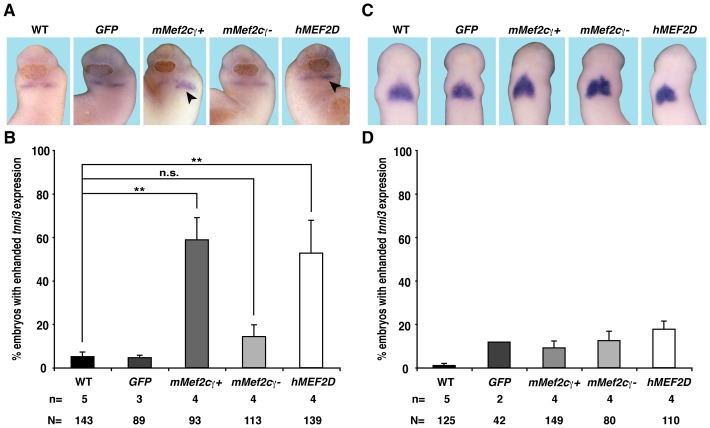
Gain of function analyzes reveals an earlier onset of cardiac differentiation. **A, C.** RNA coding for mMef2cγ+, mMef2cγ- or hMEF2D was injected unilaterally into the dorsal-vegetal blastomere at eight cell stage and *tnni3* expression was monitored at stages 24 (**A**) or 28 (**C**). Ventral views of embryos are shown. **B, D.** The percentage of embryos with enhanced expression of *tnni3* on the injected side is given. N: number of examined embryos; n: number of independent experiments; **, *p*≤0.01.

Finally, we performed gain of function experiments by injecting RNA coding for either Mef2 variant unilaterally into eight-cell stage embryos. In a first set of experiments we examined the expression of *tnni3* at stage 24 when expression of this gene commences. Injection of *mMef2c*γγ or *hMEF2D* RNA resulted in a slight increase of expression on the injected side. This was not observed in case of RNA coding for *mMef2c*γ- (Fig. 8 A, B). In contrast, we did not see any significant differences between the injected and the uninjected side when analyzing *tnni3* expression at stage 28 (Fig. 8 C, D). Taken together, these data indicate that overexpression of *mMef2c*γ+ or *hMEF2D* but not *mMef2c*γ- rather accelerates the onset of cardiac differentiation than resulting in ectopic cardiomyocyte formation.

## Discussion

Members of the Mef2 family of transcription factors are critical regulators of cardiac development in different species. However, no functional studies on *mef2* genes during cardiogenesis have been performed in *Xenopus laevis* so far. A detailed synteny analysis as well as a comprehensive comparison of cDNA sequences confirmed the correct assignment of *mef2a*, *c*, and *d* homologs in *Xenopus*. This correct assignment is certainly important for the interpretation of the cross species rescue experiments performed in this study. Surprisingly, we were not able to identify any genomic sequence or any EST coding for *mef2b* in *Xenopus* and it is very likely that *mef2b* does not exist in *Xenopus*. It is possible, however, that due to the lack of sequence information available in current public sequence databases, the assembly of the corresponding scaffold might not be complete yet. We argue, however, that as long as no *mef2b* ortholog is identified in *Xenopus*, it is likely that this gene was lost during evolution in this species.

In mice, Mef2a and Mef2c are functionally important for cardiac development as assessed by the phenotypes in genetically modified mice that lack either of these genes [17], [18]. Knock out mice of either *Mef2a* or *Mef2c* die during embryogenesis due to problems in cardiac development. In contrast, Mef2d null mice develop normally until birth. Interestingly, *mef2a* expression was recently found not to be present during cardiac development in *Xenopus* [13], whereas *mef2c* and *mef2d* were robustly expressed during cardiogenesis [8]. Our functional analyses performed in this study indicate that indeed both members are needed for proper heart formation in *Xenopus*. Moreover, based on our results, we speculate that in *Xenopus*, Mef2d has taken over the function that Mef2a has in mice. A more detailed analysis of marker genes of Mef2a-deficient mice might shed more light onto this evolutionary difference. In addition, the identification of genomic regions bound by Mef2a in mice and Mef2d in *Xenopus* might help to verify or falsify this hypothesis.

Of interest is also the observation that loss of Mef2c can be rescued by an overexpression of Mef2d but not *vice versa*. Mef2 transcription factors have been shown to form homo- and heterodimers [9], [10]. It is therefore very likely that Mef2c/Mef2d and Mef2d/Mef2d dimers or Mef2d monomers are functionally important during early cardiac development in *Xenopus*. In this scenario, Mef2c/Mef2c dimers, or Mef2c monomers, would not be similarly efficient in transcription. Our findings therefore should foster more detailed analysis of this issue not only in *Xenopus* but in mouse as well.

Although Mef2a and Mef2c knock out mice have a cardiac phenotype, the role of both genes in regulating cardiac gene expression in the CPC population has not been reported and also the expression of Mef2 proteins in the early embryo has not yet been described. However, Mef2c has been shown to be expressed in early differentiating murine ES cell cultures suggesting a possible role for Mef2c in the CPC population [28]. In line with these findings is the observation that the use of a dominant negative Mef2 protein that blocks the activity of all four members of the Mef2 family in mice inhibits cardiac differentiation in P19 cells [29]. These data clearly indicate a functional redundancy of Mef2 members in mice. Interestingly, the loss of Mef2c is additionally accompanied by an up-regulation of Mef2b. Our data shown here clearly indicate that the loss of either Mef2c or Mef2d results in early defects during *Xenopus* cardiac differentiation thus suggesting that this functional redundancy is not so prominent in *Xenopus*. This statement is also supported by the observation of a more than additive phenotype after simultaneously knocking down both Mef2c and Mef2d, which thereby implies that these two transcription factors are acting synergistically rather than redundantly. The apparent lack of Mef2b in *Xenopus* might contribute to this disparate situation as well.

Transcriptional targets of Mef2 factors that have been identified in different species include alpha cardiac myosin heavy chain, alpha cardiac actin and cardiac troponin I [30]–[32]. Notably, the expression of these genes was down-regulated in our experiments after knocking down the *mef2* genes (see Fig. 4, *myh6*, *actc1* and *tnni3*,) suggesting that their regulation by Mef2 factors is also conserved in *Xenopus*. In the case of *myh6*, a recent study identified a conserved Mef2 binding site in its promoter region [33]. Altogether, the loss and down-regulation of marker gene expression in our morphant embryos is clearly in line with what previous studies have shown. Of particular interest as a target of Mef2 factors is *Nkx2-5* [29]. Interestingly, we have not observed any down-regulation of *nkx2-5* expression in the CPC population at stage 20, but we have seen a down-regulation at the stage 28. This might suggest that *nkx2-5* is a target gene of Mef2 proteins only at later stages of development or this observation might point to diverse functions of Mef2 proteins as discussed in the next section.

The γ domain of mMef2c has been shown to function as a transcriptional repressor [12]. Our rescue experiments suggest that Mef2cγ+ but not Mef2cγ- is required for early cardiogenesis. This finding is unexpected because we found mef2γ- to be expressed higher on RNA level than the mef2γ+ isoform. Also in adult muscle and during skeletal myogenesis the γ version has been described to be predominantly expressed and to be of functional relevance [12]. Of note, we cannot exclude other functions for the mef2cγ- variant that were not tested in our study. Interestingly, overexpression of a Mef2 engrailed repressor fusion protein (MEF2/EnR) in P19 cells results in an activation of *Nkx2-5* during early phases of cardiogenesis whereas this construct inhibits cardiogenesis at later time points [29]. This is in line with our findings obtained here and implies that different splice variants of Mef2 transcription factors might be expressed during different phases of cardiac development. This has not been analyzed, at least to our knowledge, in a time and cell type specific manner during vertebrate cardiogenesis. The functional differences of two of these splice variants in our study indicate the requirement of additional expression and functional studies in the future to complete our understanding of Mef2 function during cardiogenesis. The γ domain has also been shown to be subject to regulation by phosphorylation [12] suggesting that this type of regulation occurs during early cardiac development adding another layer of complexity to Mef2 function during this time period. The fact that human MEF2D that also harbors a sequence similar to the γ domain of Mef2c is able to rescue the Mef2c MO phenotype might trigger further experiments to analyze this short stretch of amino acids in MEF2D in more detail. Finally, the discovery that Mef2d is involved in *Xenopus* cardiogenesis may also be useful as another model suitable to gain further insights into the function of Mef2d during cardiac remodeling in mice, particularly in the reactivation of a fetal gene expression program.

Of note, our experiments have additional implications as Mef2c was used as one of three components in direct reprogramming experiments to generate cardiomyocytes out of cardiac fibroblasts [25]. Our findings should therefore also foster research that examines different Mef2 splice variants and their potential roles and functions in this setting with the aim to increase the efficiency of reprogramming.

## Materials and Methods

### *Xenopus* embryos

*Xenopus laevis* embryos were obtained by *in vitro* fertilization, cultured and staged according to Nieuwkoop and Faber [34]. All procedures were performed according to the German animal use and care law (Tierschutzgesetz) and approved by the German state administration Baden-Württemberg (Regierungspräsidium Tübingen).

### Cloning

The *mMef2c* and *hMEF2D* constructs were purchased from ImaGenes GmbH and the open reading frames were subcloned into pCS2+ [35]. To obtain a rescue construct which is not targeted by the Mef2d MO, we inserted two additional silent point mutations in the Δ5′UTR *hMEF2D* by mutagenesis using the QuickChange II Site-Directed Mutagenesis Kit (Stratagene). All constructs were verified by sequencing. The *mMef2c*γ- deletion construct was generated by inverse PCR using the *mMef2c*γ+/*pCS2*+ rescue construct as template and the proof reading Phusion DNA polymerase (Finnzyme) followed by re-ligation. The primers used for the inverse PCR were: for: 5′-GAC CGT ACC ACC ACC CCT TCG A-3′; rev: 5′-GCT GAG GCT TTG AGT AGA AGG CAG G-3′. For cloning full length *mef2c* from heart enriched explants the following primers were used: Mef2c cloning for: 5′-GTT GGA GCA GAG GGG AAA AT-3′, Mef2c cloning rev: 5′-GGT ATA AGC ACA CAC ACA CTG CA-3′.

### Morpholino oligonucleotide (MO), MO specificity tests and RNA injections

All MOs were purchased from Gene Tools, LLC, OR USA, resuspended in DEPC-H_2_O and stored as aliquots at −20°C. Mef2cMO: 5′-CCA TAG TCC CCG TTT TTC TGT CTT C-3′; Mef2dMO: ′-AAT CTG GAT CTT TTT TCT GCC CAT G-3′5. For knock down approaches, we injected the MOs (10 ng) into both dorso-vegetal blastomeres of eight-cell embryos to target the presumptive heart region [27]. For lineage labeling and to identify the injected side, we co-injected 0.5 ng *GFP* RNA. Only correctly injected embryos were considered for the experiments. For control injection experiments, the standard Control MO of Gene Tools was used. Control MO: 5′-CCT CTT ACC TCA GTT ACA ATT TAT A-3′ For rescue experiments, mRNA (0.5 ng) was injected together with MO. The binding specificity of MOs was tested *in vivo* were cloned in frame with and in front of the *GFP* open reading frame in pCS2+. The indicated RNA and MO were co-injected bilaterally into two-cell stage embryos and GFP translation was monitored at stage 20 using a fluorescence microscope.

### Whole Mount *in situ* Hybridization (WMISH)

Probes for *mef2c* and *mef2d* were used as previously described [8]. Digoxigenin-labelled antisense RNA probes of analyzed genes were synthesized by restriction digestion and subsequent transcription with Sp6 or T7 RNA polymerase (Roche). To analyze marker gene expression at stages 20 and 28, MOs (10 ng) were unilaterally injected in one dorso-vegetal blastomere together with 0.5 ng *GFP* RNA. The uninjected side served as internal control. Wild type or injected embryos were fixed at +4°C in MEMFA (0.1 M MOPS, pH 7.4, 2 mM EGTA, 1 mM MgSO4, and 4% formaldehyde) at indicated stages. WMISH experiments were performed according to a standard protocol as previously described [36], [37]. BM-Purple (Roche) was used for staining. Stained embryos were then bleached with 30% H_2_O_2_. For sections, stained embryos were embedded in gelatine/albumine overnight at +4°C, sectioned using a vibratome with a thickness of 25 μm, coverslipped, and imaged with an Olympus BX60 microscope.

### RNA isolation, RT-PCR and qPCR

Total RNA was isolated from whole *Xenopus* embryos at different stages with peqGOLD RNAPure (peqLab) according to the manufacturer's protocol. To analyze *mef2c* variants in *Xenopus* cardiac tissue, the anterior-ventral part of stage 24, 28, and 32 of wild type embryos (heart-enriched regions) posterior to cement gland were dissected and total RNA was isolated using peqGOLD RNAPure (peqLab). cDNA was synthesized using random hexamers and the SuperScript II reverse transcriptase (Invitrogen). PCR was performed with the Phire Hot Start II DNA Polymerase (Thermo scientific). Primers for amplification were: gapdh_for: 5′-GCC GTG TAT GTG GAA TCT-3′; gapdh rev: 5′-AAG TTG TCG TTG ATG ACC TTT GC-3′; H4 for: 5′-CGG GAT AAC ATT CAG GGT ATC ACT-3′; H4 rev: 5′-ATC CAT GGC GGT AAC TGT CTT CCT-3′; *Xenopus* mef2c for: 5′-AGA GCG CAC GGA CTA CTG AT-3′; Xenopus_mef2c_rev: 5′-TCA CCT GTC GGT TAC GTT CA-3′; *Xenopus* mef2d for: 5′-GCA GCT TTA AAT TCC GCA AG-3; *Xenopus* rev: 5′-CGG TGT CAC TTG GCC TTT AT-3′; Quantitative PCR was performed on RNA isolated from heart-enriched regions using SYBR Green Master Mix (Fermentas) and a Roche Light Cycler 1.5. *gapdh* was used as housekeeping gene. Each sample was analyzed in triplicate. Primer pairs used were: *Xenopus* gapdh for: 5′-GCC GTG TAT GTG GAA TCT-3′; *Xenopus* gapdh rev: 5′-AAG TTG TCG TTG ATG ACC TTT GC-3′; *Xenopus* mef2cγ+ for: 5′-GCACAATATGCCTCATTCAGCC-3′; *Xenopus* mef2cγ+ rev: 5′-GGAGGAGAAACAGGTTCTGACTTG-3′; *Xenopus* mef2cγ- for: 5′-TGGCTCAGTTACTGGCTGGCAGC-3′; *Xenopus* mef2cγ-rev: 5′-TAGTACGGTCTCCCAGCTGGCTGAG-3′; *Xenopus* mef2d for: 5′AGA CCT GGC ATC CCT CTC TA-3′; *Xenopus* mef2d rev: 5′TTG CGG TTG GTT ATG TTG TT-3′.

### Western Blot

30 embryos injected with RNA were homogenized in 300 µl lysis buffer (20 mM Tris/HCl, 150 mM NaCl, 1 mM EDTA, 1 mM EGTA, 1% Triton X-100, 1 mM PMSF) and incubated on ice for 5 min. Protein samples were cleared at 13,000 rpm at 4°C for 5 min. For removal of lipids, the supernatant was mixed with 50 µl Freon followed by centrifugation at 13,000 rpm at 4°C for 5 min. The upper protein containing phase was collected. Concentration of protein samples was determined by Bradford assay with BSA as standard. Western blotting was performed according to standard procedures and proteins were visualized using a Li-COR ODYSSEY Imager. Primary antibodies were purchased from Abcam (rabbit polyclonal Mef2C, directed against residues 450 to the C-terminus of human MEF2C), Santa Cruz (mouse monoclonal anti Mef2D, H11, epitope mapping within an internal region of MEF-2D of human origin) and Serotec (γ-Tubulin, clone YL1/2). Secondary antibodies used were IRDye conjugates from Li-COR.

### Synteny analysis

For synteny analysis, genomic structure and chromosomal organization of *Mef2a*, *b*, *c* and *d* in human, mouse and *X. tropicalis* were compared using NCBI and Xenbase G Browse.

### Statistics

Data were obtained from at least three independent experiments and analyzed with statistical program GraphPad Prism. The number of embryos (N) and the number of independent experiments (n) performed for each experiment is indicated in the corresponding figures. For rescue experiments, embryos of the same batch were evaluated upon injection either with either MO or MO along with the RNA of interest. The nonparametric Mann-Whitney rank sum test was used to determine statistical differences. A *p* value of ≤0.05 was considered to be significant.

## Supporting Information

Figure S1 **Spatio-temporal expression of** ***mef2c*** **and** ***mef2d*** **in** ***Xenopus*** **.** **A.** Temporal expression of *mef2c*. *mef2c* is maternally supplied. *Mef2c* embryonic expression starts at stage 9 and increases until stage 40. *gapdh* was used as loading control. –RT serves as negative control. ***B*** *.* Temporal expression of *mef2d*. *mef2d* is maternally supplied. *Mef2d* embryonic expression starts at stage 12. *H4* was used as loading control. –RT serves as negative control. **C–j**. Spatial expression of *mef2c*. C. Anterior view with the dorsal side to the top. **c**. Sagittal section. **D, F, H, J**. Lateral views with anterior to the right. **E, G, I, K**. Ventral views with anterior to the top. Black arrowheads indicate the expression in the FHF, the red arrowhead highlights *mef2c* transcripts at the lateral sides of the SHF. **C.** Parasagittal section. **d, f, h, j.** Transverse sections. Black arrowheads indicate cardiac expression; the arrowhead in h shows *mef2c* expression in the endocardium, the black arrow in the myocardium. **L–s.** Spatial expression of *mef2d*. **L.** Anterior view with the dorsal side to the top. **l.** Sagittal section. **M, O, Q, S.** Lateral views with anterior to the right. **N, P, R, T**. Ventral views with anterior to the top. **m, o, q, s.** Transverse sections. White arrows indicate *mef2d* expression in cardiac progenitor cells. The white arrowhead indicates cardiac cells with low *mef2d* expression. Black arrows indicate *mef2d* expression in the myocardium, black arrowheads show *mef2c* expression in the first heart field (FHF). St: stage
(TIF)

Figure S2 ***In vivo*** **MO specificity test.** Two-cell stage embryos were bilaterally injected and GFP fluorescence was monitored at stage 20. MO binding sites of *Xenopus*, mouse and human are indicated. Red letters indicate different bases in the MO binding sites, green letters indicate the ATG start codon. Upper panels show the light view, lower panels provide the fluorescent view. **A.** GFP fluorescence was observed upon injection of *mef2c-GFP* together with Control MO but not with Mef2c MO. Neither *mMef2c-GFP* nor *hMEF2D-GFP* were targeted by Mef2c MO. **B.** GFP expression was observed after the injection of Control MO. Co-injection of *xmef2d-GFP* and Mef2d MO led to an inhibition of GFP expression. Neither the expression of *hMEF2D-GFP* nor *mMef2c-GFP* was influenced by Mef2d MO.
(TIF)

Table S1 Gene abbreviations used in Figure 1.
(PDF)
