# Supplementary figures and images for: Comparative Analysis Reveals Distinct and Overlapping Functions of Mef2c and Mef2d during Cardiogenesis in Xenopus laevis
Source: PLoS One. 2014 Jan 28;9(1):e87294. doi: 10.1371/journal.pone.0087294 (PMC3904989; doi:10.1371/journal.pone.0087294)

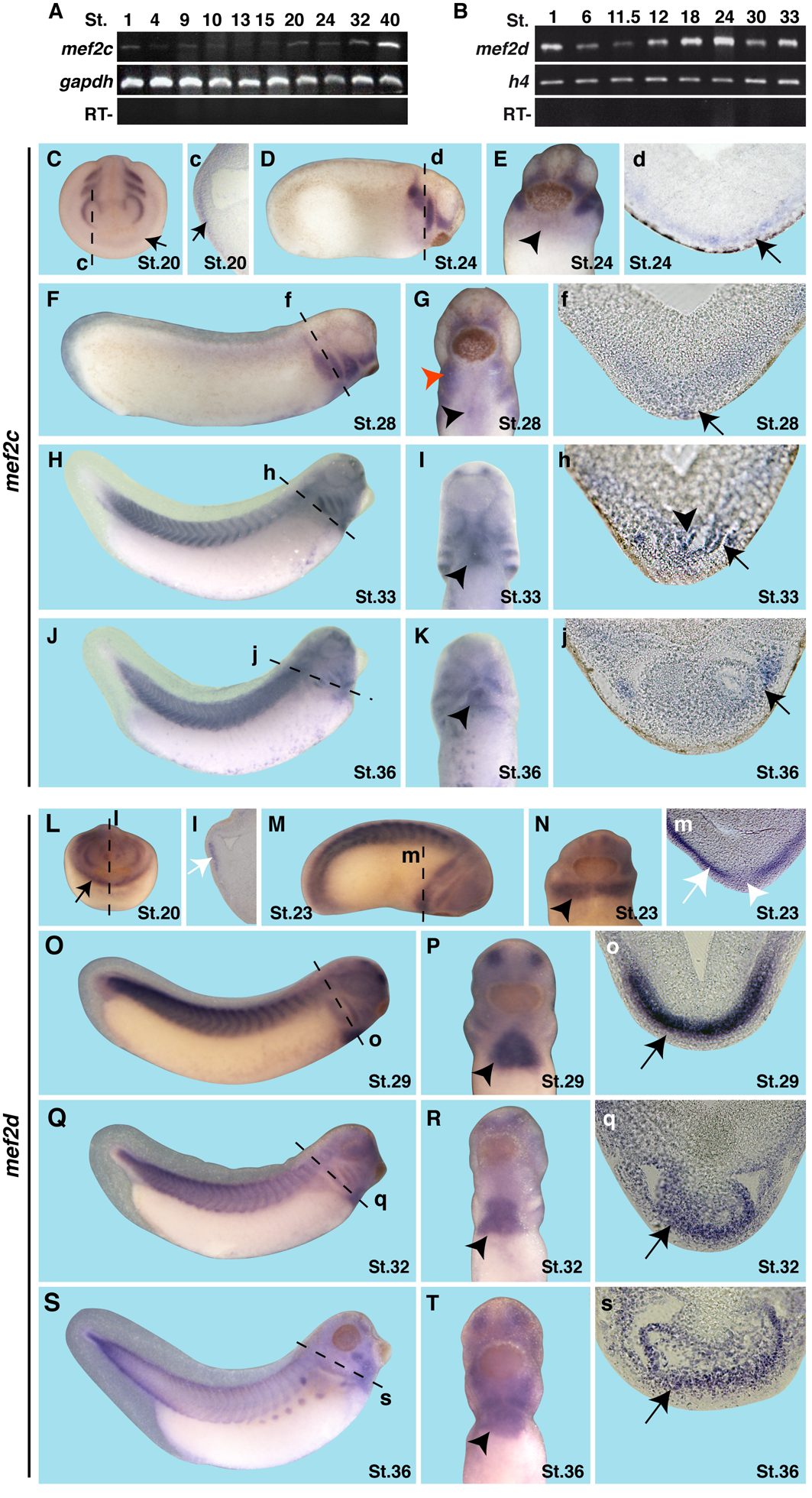

Supplement: Figure S1 — Spatio-temporal expression of mef2c and mef2d in Xenopus . A. Temporal expression of mef2c. mef2c is maternally supplied. Mef2c embryonic expression starts at stage 9 and increases until stage 40. gapdh was used as loading control. –RT serves as negative control. B . Temporal expression of mef2d. mef2d is maternally supplied. Mef2d embryonic expression starts at stage 12. H4 was used as loading control. –RT serves as negative control. C–j. Spatial expression of mef2c. C. Anterior view with the dorsal side to the top. c. Sagittal section. D, F, H, J. Lateral views with anterior to the right. E, G, I, K. Ventral views with anterior to the top. Black arrowheads indicate the expression in the FHF, the red arrowhead highlights mef2c transcripts at the lateral sides of the SHF. C. Parasagittal section. d, f, h, j. Transverse sections. Black arrowheads indicate cardiac expression; the arrowhead in h shows mef2c expression in the endocardium, the black arrow in the myocardium. L–s. Spatial expression of mef2d. L. Anterior view with the dorsal side to the top. l. Sagittal section. M, O, Q, S. Lateral views with anterior to the right. N, P, R, T. Ventral views with anterior to the top. m, o, q, s. Transverse sections. White arrows indicate mef2d expression in cardiac progenitor cells. The white arrowhead indicates cardiac cells with low mef2d expression. Black arrows indicate mef2d expression in the myocardium, black arrowheads show mef2c expression in the first heart field (FHF). St: stage (TIF) [file pone.0087294.s001.tif]

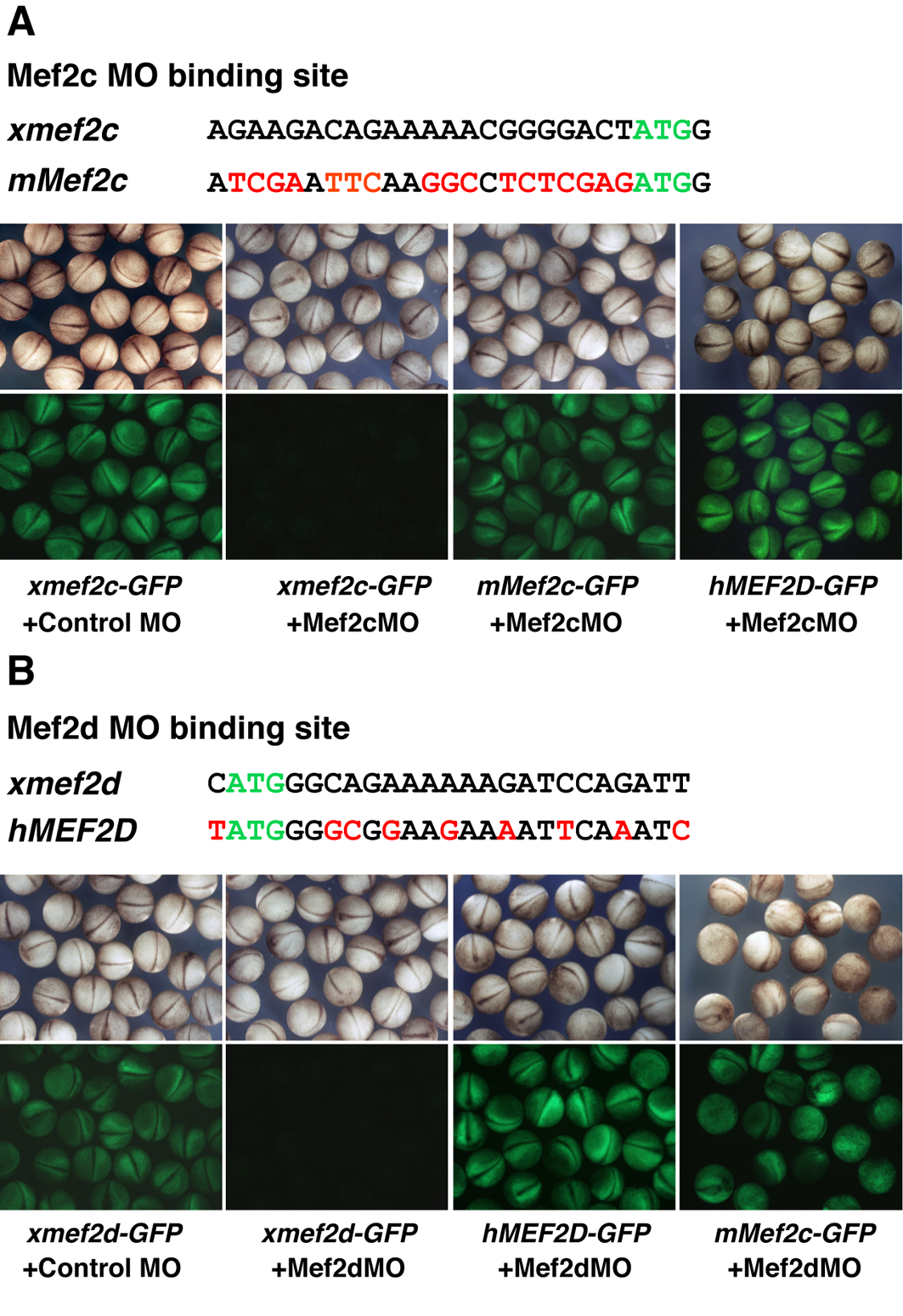

Supplement: Figure S2 — In vivo MO specificity test. Two-cell stage embryos were bilaterally injected and GFP fluorescence was monitored at stage 20. MO binding sites of Xenopus, mouse and human are indicated. Red letters indicate different bases in the MO binding sites, green letters indicate the ATG start codon. Upper panels show the light view, lower panels provide the fluorescent view. A. GFP fluorescence was observed upon injection of mef2c-GFP together with Control MO but not with Mef2c MO. Neither mMef2c-GFP nor hMEF2D-GFP were targeted by Mef2c MO. B. GFP expression was observed after the injection of Control MO. Co-injection of xmef2d-GFP and Mef2d MO led to an inhibition of GFP expression. Neither the expression of hMEF2D-GFP nor mMef2c-GFP was influenced by Mef2d MO. (TIF) [file pone.0087294.s002.tif]
