# Supplementary material for: Comparative Analysis Reveals Distinct and Overlapping Functions of Mef2c and Mef2d during Cardiogenesis in Xenopus laevis
Source: PLoS One. 2014 Jan 28;9(1):e87294. doi: 10.1371/journal.pone.0087294 (PMC3904989; doi:10.1371/journal.pone.0087294)
Supplement: Table S1 — Gene abbreviations used in Figure 1. (PDF) [file pone.0087294.s003.pdf]

Table S1 *Gene abbreviations used in Figure 1*

Adamts17: ADAM metallopeptidase with thrombospondin type 1 motif, 17

Ankrd32: ankyrin repeat domain 32

Apoa1bp: apolipoprotein A-I binding protein

Armc6: armadillo repeat containing 6

Asb7: ankyrin repeat and SOCS box containing 7

Ccnh: cyclin H

Cct3: chaperonin containing TCP1, subunit 3 (gamma)

Cetn3: centrin, EF-hand protein, 3; Tmem161b: transmembrane protein 161B

Chsy1: chondroitin sulfate synthase 1

Cox7c: cytochrome c oxidase subunit VIIc

Edil3: EGF-like repeats and discoidin I-like domains 3

Fam169b: family with sequence similarity 169, member B

Fam172a: family with sequence similarity 172, member A

Gata2a: Gata-binding protein 2a

Gpatch4: G patch domain containing 4

Gpr98: G protein-coupled receptor 98

Hapln4: hyaluronan and proteoglycan link protein 4

Igf1r: insulin-like growth factor 1 receptor

Iqgap3: IQ motif containing GTPase activating protein 3

Lass3: ceramide synthase 3

Lins: lines homolog

Lmna: lamin A/C

Lrrc28: leucine rich repeat containing 28

Lrrk1: leucine-rich repeat kinase 1

Lysmd3: LysM, putative peptidoglycan-binding, domain containing 3

Lysmd4: LysM, putative peptidoglycan-binding, domain containing 4

Mau2: MAU2 chromatid cohesion factor homolog

Mblac2: metallo-beta-lactamase domain containing 2

Mctp1: multiple C2 domains, transmembrane 1

Mef2bnb: MEF2B neighbor

Ncan: neurocan**;** Nr2c2ap: nuclear receptor 2C2-associated protein

Pcsk6: proprotein convertase subtilisin/kexin type 6

Pgpep 1l: pyroglutamyl-peptidase I-like

Pmf1: polyamine-modulated factor 1

Polr3G: polymerase (RNA) III (DNA directed) polypeptide G

Rab25: RAB25, member RAS oncogene family

Rasa1: RAS p21 protein activator (GTPase activating protein) 1

Rfxank: regulatory factor X-associated ankyrin-containing protein

Rhbg: Rh family, B glycoprotein (gene/pseudogene)

Sema4a: sema domain, immunoglobulin domain (Ig), transmembrane domain (TM) and short cytoplasmic domain, (semaphorin) 4A

Slc25a42: solute carrier family 25, member 42

Smg5: smg-5 homolog, nonsense mediated mRNA decay factor (C. elegans)

Sugp1: SURP and G patch domain containing 1

Synm: synemin, intermediate filament protein

Tm6sf2: transmembrane 6 superfamily member 2

Tmem161a: transmembrane protein 161A

Ttc23: tetratricopeptide repeat domain 23

Ttc24: tetratricopeptide repeat domain 24

Ubqln4: ubiquilin 4
